# Mitigation of ultraviolet‐induced erythema and inflammation by *para*‐hydroxycinnamic acid in human skin

**DOI:** 10.1111/ics.13002

**Published:** 2024-08-13

**Authors:** William P. Janson, Laurie E. Breyfogle, John C. Bierman, Zhi Yan Chew, Matthew C. Ehrman, John E. Oblong

**Affiliations:** ^1^ The Procter & Gamble Company Cincinnati Ohio USA; ^2^ Procter & Gamble International Operations (SA) Singapore Branch Singapore Singapore

**Keywords:** environmental stressors, inflammation, niacinamide, *p*‐hydroxycinnamic acid, senescence associated secretory phenotype, skin barrier

## Abstract

**Objective:**

To evaluate whether *p*‐hydroxycinnamic acid (pHCA) alone and in combination with niacinamide (Nam) can mitigate UV‐induced erythema, barrier disruption, and inflammation.

**Methods:**

Three independent placebo‐controlled double‐blinded studies were conducted on female panellists who were pretreated on sites on their backs for 2 weeks with skin care formulations which contained 0.3% or 1% pHCA with 5% Nam, 1% pHCA alone, 1.8% octinoxate, or control formula. Treated sites were then exposed to 1.5 minimal erythemal dose (MED) solar simulated radiation (SSR) and had chromameter and expert grading measures for erythema, barrier integrity via TEWL, and the skin surface IL‐1RA/IL‐1α inflammatory biomarkers isolated from D‐Squame tapes.

**Results:**

Across the three independent studies, pHCA alone or in combination with Nam showed a significant mitigation of UV‐induced erythema, barrier disruption, and levels of the surface inflammatory biomarkers IL‐1RA/IL‐1α. The cinnamate analogue Octinoxate did not replicate the effects of pHCA.

**Conclusion:**

The study results show that pHCA alone or in combination with Nam can mitigate UV‐induced damage to skin. These include mitigation of UV‐induced erythema as measured by instrument and expert grade visualization. Additionally, pHCA with Nam protected damage to the barrier and reduced the induction of the SASP‐related surface inflammatory biomarker IL‐1RA/IL‐1α. The inability of Octinoxate to have any protective effect and the detection of low levels of pHCA on skin surface after 24 h of application supports that these effects are based on a biological response to pHCA. These findings add to the body of evidence that pHCA alone or in combination with Nam can enhance the skin's biological response to UV‐induced damage. This supports pHCA can potentially impact aging and senescence, thereby maintain skin's functionality and appearance.

## Graphical abstract

*para‐*Hydroxycinnamic acid (pHCA) can dose dependently mitigate the negative impact of UV exposure on erythema, inflammatory response, and barrier disruption via a biological response.

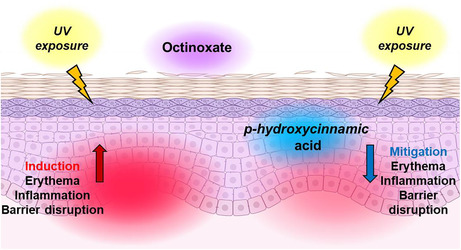

## RÉSUMÉ

**Objectif:**

Évaluer si l'acide *p*‐hydroxycinnamique (p‐hydroxycinnamic acid, pHCA) seul et en association avec le niacinamide (Nam) peut atténuer l'érythème induit par les UV, la rupture de la barrière et l'inflammation.

**Méthodes:**

Trois études en double aveugle, contrôlées par placebo et indépendantes, ont été menées auprès de femmes panélistes ayant reçu un traitement préalable sur le dos pendant deux semaines avec des formulations de soins cutanés contenant 0.3% ou 1% de pHCA avec 5% de Nam, 1% de pHCA seul, 1.8% d'octinoxate ou une formule témoin. Les sites traités ont ensuite été exposés à une dose érythémateuse minimale (MED) de 1.5 de radiation solaire simulée (SSR) et ont été évalués à l'aide de mesures par chromamètre et de cotations par des experts concernant l'érythème, l'intégrité de la barrière via la perte insensible en eau (TEWL), et les biomarqueurs inflammatoires de la surface cutanée IL‐1RA/IL‐1 α isolés à partir de bandes D‐Squame.

**Résultats:**

Dans les 3 études indépendantes, le pHCA seul ou en association avec le Nam a montré une atténuation significative de l'érythème induit par les UV, de la perturbation de la barrière et des taux des biomarqueurs inflammatoires de surface IL‐1RA/IL‐1α. L'analogue du cinnamate, l'octinoxate, n'a pas reproduit les effets du pHCA.

**Conclusion:**

Les résultats des études montrent que le pHCA seul ou en association avec le Nam peut atténuer les dommages cutanés induits par les UV. Ceux‐ci comprennent l'atténuation de l'érythème induit par les UV, mesuré par instrument et une visualisation de qualité experte. En outre, le pHCA en association avec le Nam a protégé contre les dommages de la barrière et a réduit l'induction du biomarqueur inflammatoire de surface lié à la SASP IL‐1RA/IL‐1α. L'incapacité de l'octinoxate à avoir un effet protecteur, ainsi que la détection de faibles niveaux de pHCA à la surface de la peau après 24 heures d'application, confirme que ces effets sont basés sur une réponse biologique au pHCA. Ces résultats viennent s'ajouter à l'ensemble des preuves montrant que le pHCA seul ou en association avec le Nam peut améliorer la réponse biologique de la peau aux dommages induits par les UV. Cela soutient l'hypothèse que le pHCA peut avoir une incidence potentielle sur le vieillissement et la sénescence, maintenant ainsi la fonctionnalité et l'aspect de la peau.

## INTRODUCTION

The skin is the largest continuous human organ and one of its primary functions is to protect the body from exposure to damaging external stressors such as solar radiation, chemicals, pollution, and particulate matter. Like any organ, skin is susceptible to aging and this process can be accelerated by cumulative acute microdamage from environmental stress exposure. This premature aging of skin leads to cellular and structural changes that accumulates over time and ultimately affects skin's appearance, functionality, and homeostatic state. Thus, it is of interest to understand these changes in order to identify mechanistic intervention targets that would impact premature aging and maintain skin's health and appearance.

The impact of environmental stressors on human skin health has been studied, particularly on solar radiation since it is believed that ultraviolet (UV) exposure can cause ~80% of premature aging [1, 2]. Mechanistically the initial impact on skin by environmental stressors is the generation of free radicals and reactive oxygen species (ROS) which cause DNA damage, protein structure and enzymatic activity alteration, and formation of lipid peroxides [3]. In the case of UV exposure, a classical sign of damage induction is the rapid elevation of underlying erythema due in part to the synthesis and release of inflammatory factors to signal recruitment of an innate immune infiltrate as well as capillary damage and leakiness [4, 5]. The efficiency of repair from this damage declines with age and this leads to an accumulation of damaged cellular debris, altered gene expression patterns, and changes in cellular and tissue function [6]. Thus, identifying technologies that can mitigate or reduce this damage from environmental stressors is proposed as a mechanistic approach to impact premature aging.

The plant kingdom is a rich source of diverse chemistries which plants synthesize for development and reproduction as well as help defend against UV radiation, pathogens, parasites, and predators called phytochemicals. Phenolics are one such class of chemicals that have been characterized as natural antioxidants with potential benefits to human health by scavenging free radicals or reducing activity [7, 8]. pHCA (aka *p*‐coumaric acid) is a phenolic derivative of cinnamic acid and has been reported to be an antioxidant and a free radical scavenger [9]. Additionally, pHCA has been reported to have positive benefits in oxidative stress‐related diseases, including skin aging and inflammation [10].

Relative to pHCA effects on UV‐induced skin damage, it has been previously reported that pretreatment of Fitzpatrick Types III and IV subjects with 1.5% pHCA can mitigate UV‐induced erythema [11]. To increase our understanding of pHCA's mitigating effects against UV damage, we evaluated pHCA alone and in combination with niacinamide (Nam; vitamin B_3_). Nam was identified for combination testing since it is known to have antioxidant and anti‐inflammatory properties as well as providing protection from UV‐induced damage to human skin [12, 13, 14]. Our studies utilized panellist's backs as treatment sites due to the surface area and number of treatment groups per individual. Additionally, our studies tested for pHCA dose response sensitivity as well as a stoichiometric comparison against Octinoxate, a sunscreen that shares structurally similarity to pHCA. Mechanistically, this comparison was intended to confirm any protective effect by pHCA is not due to a false sunscreen effect from residual material on the skin's surface. Finally, we also assessed the impact of pHCA on mitigating UV‐induced levels of the inflammatory biomarker ratio of IL‐1RA/IL‐1α.

We present here a body of evidence that pretreatment of human skin with pHCA can mitigate damage induced by the environmental stressor UV in a dose dependent manner and that the combination of pHCA with Nam further strengthened the degree of mitigation. Since the cinnamate core structure is known to absorb UV radiation we show that Octinoxate, a cinnamate‐based commercial sunscreen molecule that has structural similarities to pHCA, had no mitigation effect. Finally, quantitative analysis of surface residue of pHCA after a single or double application measured low levels relative to UV absorption potential, supporting that pHCA's impact on mitigating UV‐induced damage is via biological processes. We propose that pHCA can mitigate UV‐induced damage to skin, thereby delay premature aging and help maintain skin's functionality and appearance.

## MATERIALS AND METHODS

### UV challenge studies

Three independent double‐blinded placebo‐controlled studies in healthy female subjects, ages 25–60, with Fitzpatrick Skin Types II–III were performed. Study 1 was performed from April 22nd through May 29th, 2013, at Consumer Product Testing Company, Fairfield, NJ. Study 2 was performed from May 13th through June 14th, 2019, at Eurofins CRL Inc., Piscataway, New Jersey. Study 3 was performed from September 29th through December 18th, 2020, at Eurofins CRL Inc., Piscataway, New Jersey. Due to the cosmetic nature of this study, use of non‐regulated test articles, low risk to study subjects (normal expectation) an IRB was not utilized for review/approval for Study 1 or 2. This decision conformed to the respective Sponsor's SOP on IRB review. Study 3 protocol was reviewed and approved by IntegReview IRB (Austin, TX, USA).

The studies employed a randomized, complete block design, in which treatments were evaluated on separate 3 × 3 cm sites in the middle region of subjects' backs. Good clinical practices were followed, and all subjects gave their informed consent for inclusion before they participated in the study. In all studies there was a 1‐week wash‐out period, during which time each subject's minimal erythemal dose (MED) was determined for calculating the UV exposure dose to deliver after 2 weeks of treatment. MED references the lowest exposure time that produces minimally perceptible erythema 20–24 h after irradiation. For Study 1 the single UV induction dose was 1.5 times the MED. For Study 2 and 3, the single UV induction dose was based on the MED and a multiplier, where the multiplier values ranged from 1.5 to 2.5 (with 0.25 increments). Wash‐out was followed by 2 weeks of daily test product application (3 μL/cm^2^) conducted at the respective study facility before a single controlled UV exposure of 1.5 MED or target a* value ≥12.0 on each site. In Study 1 and 2, daily product applications were continued on the day of 1.5+ UV exposure (following the exposure event) and at least the next 3consecutive days. In Study 3, no product was applied after the 1.5+ MED SSR exposure. At evaluation visits, all measurements and imaging were completed before treatments were applied. Measurements were obtained in a controlled temperature and humidity environment (70 ± 2°F and 30%–45% relative humidity). Measurements taken on each treatment site included live visual redness grading by 2 trained graders, Chromameter, AquaFlux, and full back images at baseline, pre‐UV exposure, and 1–3 continuous days post‐UV exposure.

For the by‐day statistical analysis, for Study one, the change from pre‐UV baseline (week 2) was the primary measure for the analysis. This parameter was statistically analysed using a mixed model Repeated Measures Analysis of Covariance (ANCOVA) with treatment, side, site, and time as factors, baseline, age, Fitzpatrick skin type as covariates, and treatment‐by‐time interaction. A compound symmetry correlation structure was assumed for the repeated measurements taken from each subject. Homogeneity of Variance was checked at each time point using the Levene test to determine equal or unequal treatment variances. For Study 2 and 3, the change from pre‐UV baseline (week 2) was statistically analysed using an ANCOVA model for the by‐day results with treatment, side, site and cohort as factors, and baseline as a covariate. For all three studies, model assumptions were assessed to check the robustness of the conclusions.

### Extraction and quantitation of IL‐1RA and IL‐1α from D‐squame tapes

In Study 1, four separate 22 mm diameter D‐Squame® tape strips (Clinical and Derm, Dallas, TX, USA) were used to collect surface material from the control, 5% Nam, or 1% pHCA +5% Nam treated sites at the end of pretreatment prior to UV exposure and 16 days post‐UV exposure. Total protein was extracted from the tapes and analysed for IL‐1RA and IL‐1α via ELISA as previously described [15]. Repeated measures ANOVA was used for statistical analysis, modelling the original baseline as a covariate on original values and change from baseline.

### Quantitation of pHCA residual surface levels from D‐squame tapes

A 30 μL aliquot from 1% pHCA study formula product was applied to the left forearm of two volunteers at three different locations (each location was 3 × 5 cm) and allowed to air dry for 10 min. Subjects performed normal day‐to‐day procedures, including showering the following day prior to tape strip collection. The three locations on the left forearm of each subject were labelled as Site A (upper forearm), Site B (mid forearm) and Site C (close to wrist). 22 mm diameter D‐Squame tape strip (Clinical and Derm, Dallas, TX, USA) samples (10 sequential tape strips from the same spot) were collected from Site C immediately after a 10 min dry time and from Site B after 24 h. A second 30 μL application of the pHCA formulation was applied to Site A and allowed to air dry for 10 min and then 24 h later 10 consecutive tape strip samples were collected from the same spot. A 30 μL aliquot of a SC 99 Vehicle Control was applied to the right forearm of each subject at a site close to the wrist (Site D), allowed to dry for 10 min and then 10 sequential tape strips were taken from the same spot on this site. The tape strips were transferred to 2 mL polypropylene tubes along with a 1 mL aliquot of a 50/50 methanol/water extraction solution. The samples were sonicated for 15 min, the extraction solution was transferred to a second 2 mL polypropylene tube and an aliquot of the extract was diluted with 50/50 water/MeOH. Site A and B samples were diluted 5‐fold and Site C samples were diluted 10‐fold. The Site D samples (control) were diluted 2‐fold. An aliquot of each diluted sample was spiked with a 20 μL of a 20 μg/mL of a stable‐isotope Internal Standard (pHCA‐d_4_) solution. Isocratic reversed‐phase LC/MS/MS using multiple reaction monitoring in the negative ionization electrospray mode was used to analyse the standards, QCs, and sample extracts. The peak area ratio (area of pHCA peak/Area of Internal Standard peak) for the standards versus pHCA concentration (10–10 000 ng/mL in 50/50 water/MeOH) were fitted by a quadratic 1/*x* ^2^ standard curve. The levels of pHCA in the QC and Study Samples were determined by interpolation from the linear regression curve.

## RESULTS

### Topical treatment with pHCA with or without Nam can mitigate UV‐induced erythema

**FIGURE 1:**
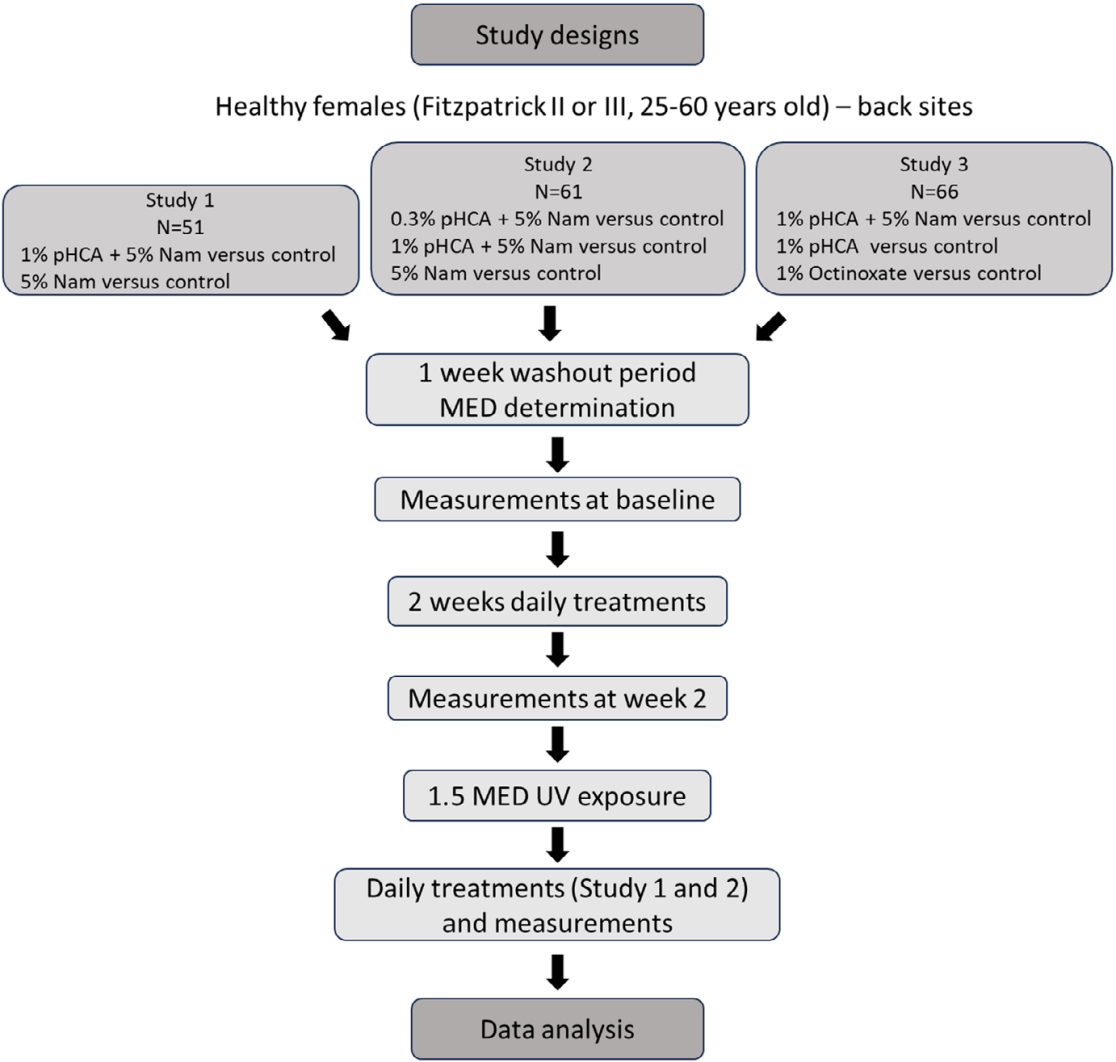
Flowchart outlining the study design of the three independent UV exposure treatment studies. Measurements included Chromameter L*a*b readings, erythema expert grading, AquaFlux TEWL, digital imaging, and D‐squame tape strip collection for biomarker analysis (Study 1 only).

To assess the impact of pretreatment with pHCA on protecting against acute UV‐induced damage to skin, three independent double‐blinded, placebo‐controlled UV challenge studies were fielded. A flowchart outlining the overall study design of these studies is shown in Figure 1. The studies required controlled on‐site daily application (minus weekends) of an oil‐in‐water emulsion skin care formulation containing pHCA, Nam, Octinoxate, or a combination of pHCA with Nam and the measurements taken included chromameter L*a*b readings and expert grading of redness to assess erythema changes, AquaFlux TEWL to measure barrier integrity, digital imaging to visualize erythema differences, and D‐squame tape strip collection to quantitate changes in the inflammatory biomarker ratio IL1‐RA/IL‐1α (Study 1 only). After 2 weeks of product application, all sites were exposed to a single 1.5–2.5 MED dose of solar simulated radiation (SSR; UVA and UVB proportion of 89.5:10.5). After exposure, in Study 1 and 2 panellists continued to have product applied for an additional 3 days; in Study 3 product application was limited to the pre‐UV exposure period. Study measures were collected pre‐UV exposure and at least three continuous days post‐UV exposure. In Study 1 D‐Squame tape strips were collected 16 days after UV exposure.

**FIGURE 2:**
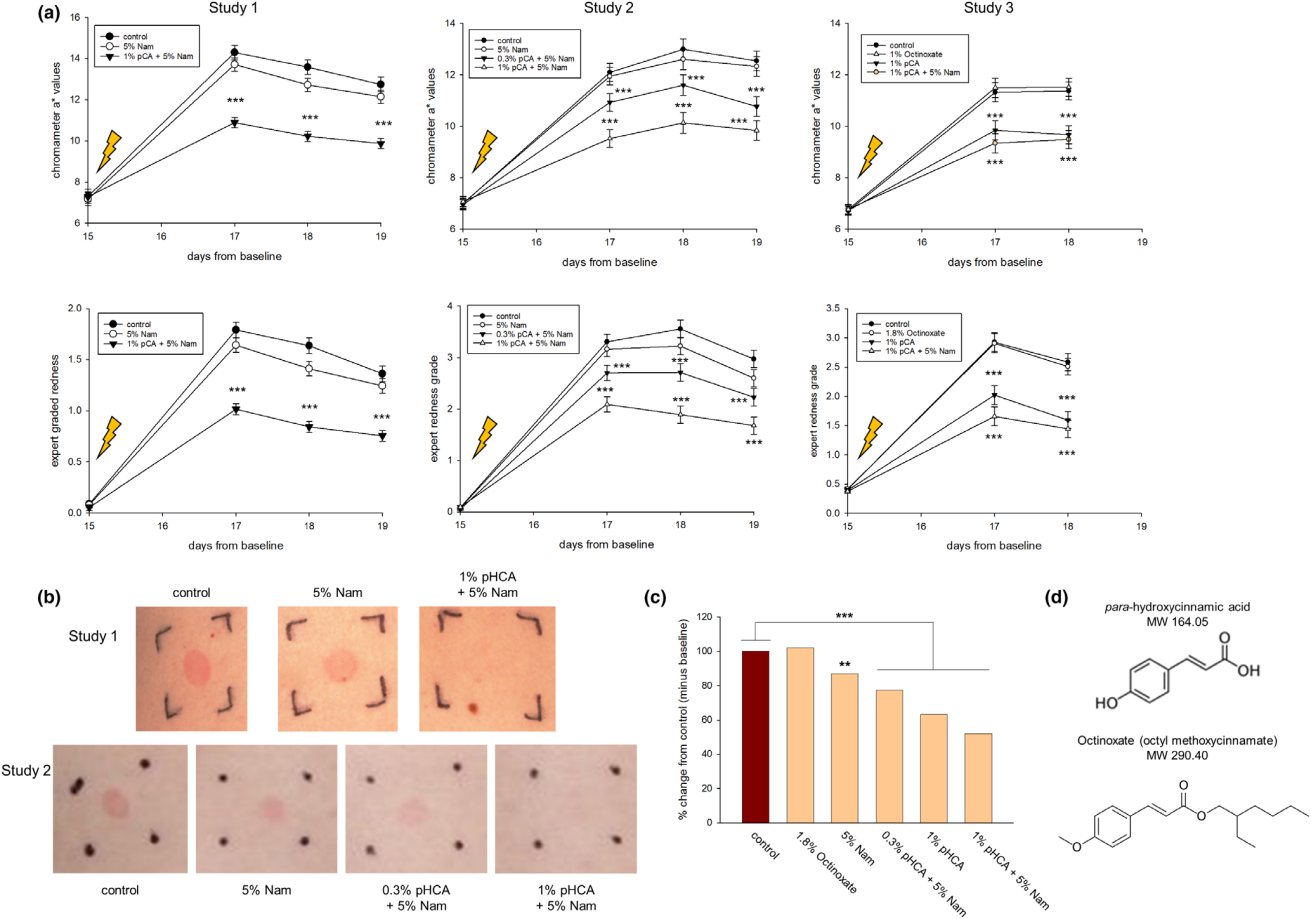
Treatment with pHCA with or without Nam reduced erythema induced by UV exposure using solar simulated radiation (SSR). Female panellists were treated on a 3 × 3 cm site on their backs for 14 days with an emulsion vehicle control and an emulsion containing 5% Nam. After pretreatment phase, the treated sites were exposed to a 1.5 minimal erythema dose (MED) with SSR (UVA and UVB proportion of 89.5:10.5). (a) Chromameter a* value readings were collected at the end of the pretreatment phase prior to SSR exposure (arrow) and at 2, 3, and 4 days post‐UV exposure. In Study 1 treatment with 1% pHCA +5% Nam showed a significant reduction in a* values compared to vehicle control at all three time points post SSR exposure. Visual grading for erythema utilizing a 6‐point redness scale to calculate redness between treatment groups showed a corresponding significant reduction in redness. In Study 2 treatment with 0.3% pHCA +5% Nam or 1% pHCA +5% Nam showed significant reductions in a* values compared to vehicle control at all three time points post‐UV exposure. Visual grading for erythema showed a corresponding significant reduction in redness. In Study 3 treatment with 1% pHCA or 1% pHCA +5% Nam showed significant reductions in a* values compared to vehicle control at both time points post‐UV exposure and visual grading showed a corresponding significant reduction in redness. 1.8% Octinoxate had no effect on mitigating SSR‐induced erythema. (b) A representative set of digital colour images from Study 1 (vehicle control, 5% Nam, or 1% + 5% Nam) and Study 2 (vehicle control, 5% Nam, 0.3% pHCA +5% Nam, or 1% pHCA +5% Nam) 2 days after UV exposure. (c) Comparison of the relative percent change in a* values compared to vehicle across all three studies shows that 5% Nam, 0.3% pHCA +5% Nam, 1% pHCA, and 1% pHCA +5% Nam significantly reduce erythema 2 days post‐UV exposure by 13%, 23%, 27%, and 48%, respectively. (d) Chemical structures and molecular weights of pHCA and Octinoxate. (**p < 0.01, ***p < 0.001, ±SEM). SSR, solar simulated radiation.

In Study 1, pretreatment with 1% pHCA +5% Nam showed a significant reduction in a* values compared to vehicle control at all three time points post‐UV exposure (Figure 2a). There was no significant impact of any treatments on L* or b* chromameter values (data not shown). Visual grading for erythema utilizing a 6‐point redness scale to calculate redness between treatment groups showed a corresponding significant reduction in redness (Figure 2a). In Study 2 treatment with 0.3% pHCA +5% Nam or 1% pHCA +5% Nam showed significant reductions in a* values compared to vehicle control at all three time points post SSR exposure (Figure 2a). Visual grading for erythema showed a corresponding significant reduction in redness. Across both metrics of a* values and expert grading, 1% pHCA +5% Nam was significantly better than 0.3% pHCA +5% Nam at mitigating overall redness, supporting a dose response differential. In Study 3 treatment with 1% pHCA or 1% pHCA +5% Nam showed significant reductions in a* values compared to vehicle control at both time points post‐UV exposure and visual grading showed a corresponding significant reduction in redness (Figure 2a). 1.8% Octinoxate had no effect on mitigating SSR‐induced erythema. A representative set of digital colour images from Study 1 (vehicle control, 5% Nam, or 1% + 5% Nam) and Study 2 (vehicle control, 5% Nam, 0.3% pHCA +5% Nam, or 1% pHCA +5% Nam) 2 days after UV exposure show the visual differentials across treatments (Figure 2b). To better compare across studies, the relative percent change in a* values compared to vehicle across all three studies shows that 5% Nam, 0.3% pHCA +5% Nam, 1% pHCA, and 1% pHCA +5% Nam significantly reduce erythema 2 days post‐UV exposure by 13, 23, 27, and 48%, respectively (Figure 2c).

### Topical treatment with pHCA can protect barrier integrity and reduce the ratio of the skin surface inflammatory biomarkers IL‐1RA/IL‐1α from SSR induction

**FIGURE 3:**
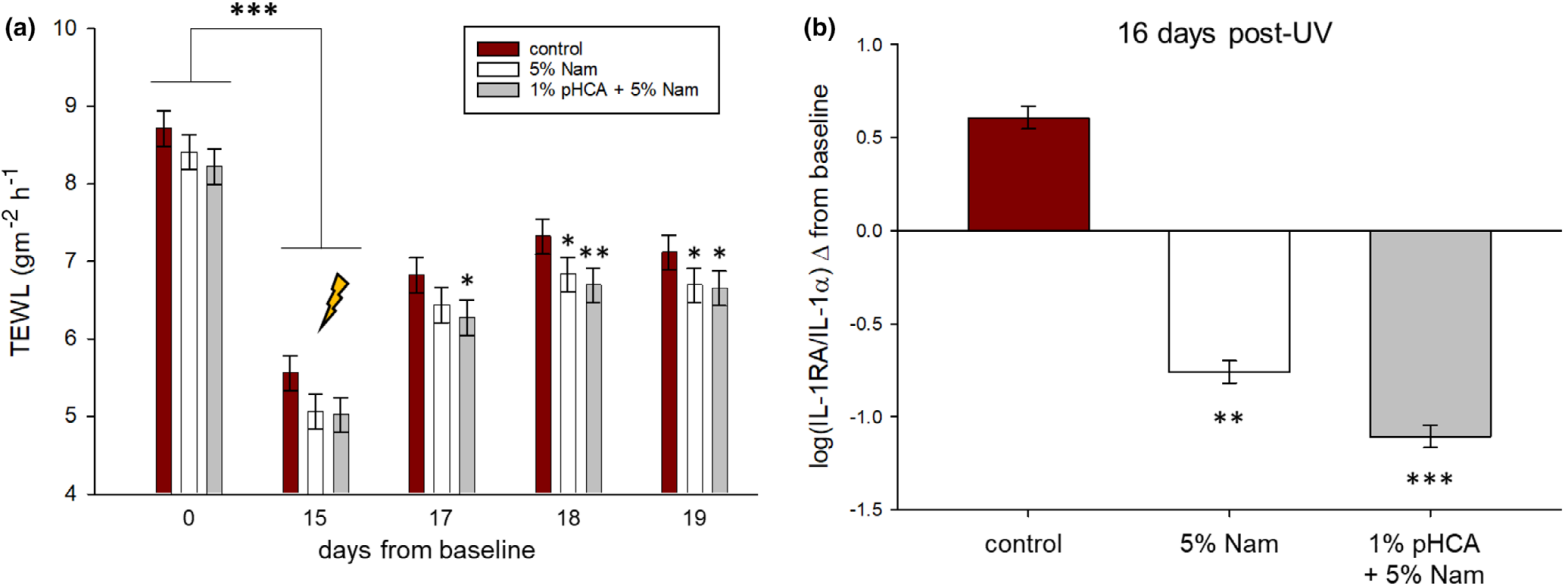
Topical pHCA combined with niacinamide partially protected barrier integrity and reduced levels of skin surface inflammatory biomarkers from UV exposure in Study 1. (a) TEWL measurements using an Aquaflux instrument were collected at the end of the pretreatment phase prior to SSR exposure (day 15, arrow) and at 2, 3, and 4 days post SSR exposure. All three treatment groups significantly lowered TEWL measures from day 0 to day 15. 1% pHCA +5% Nam had significant effects on maintaining barrier integrity on three consecutive days post SSR exposure. (b) The ratio of the skin surface inflammatory biomarkers IL‐1RA/IL‐1α induced by solar simulated radiation (SSR). Four separate D‐Squame® tape strips were collected from each site after pretreatment phase and 16 days after UV exposure. Quantitation of extracted IL‐1RA/IL‐1α protein ratio showed a significant reduction by 1% pHCA +5% Nam and 5% Nam versus control when comparing changes from day 15 pre‐SSR exposure levels. (*p < 0.05, **p < 0.01, ***p < 0.001, ±SEM). SSR, solar simulated radiation.

TEWL values across all three studies were collected at varying time points both before and after UV exposure. In Study 1 1% pHCA +5% Nam showed a lower TEWL change from pre‐UV exposure across 3 days after UV exposure (Figure 3a). 5% Nam also showed some a lowering of TEWL changes albeit to a weaker degree (Figure 3a). TEWL measurements in Study 2 and 3 showed varying degrees of barrier integrity protection by pHCA containing treatment groups, ranging between numerically to significantly better compared to control (data not shown). To measure the effect of 1% pHCA +5% Nam treatment on a UV‐induced inflammatory response, D‐Squame® tape strips were used to collect skin surface material for quantitation of the inflammatory biomarkers IL‐1RA and IL‐1α. Both 5% Nam and 1% pHCA +5% Nam showed significantly lower levels of the ratio compared to control sites after UV exposure (Figure 3b). These data support the erythema measures where treatment with either 5% Nam or 1% pHCA +5% Nam can partially mitigate the inflammatory response induced by UV.

### Skin surface levels of topically applied pHCA does not accumulate to a meaningful level

**FIGURE 4:**
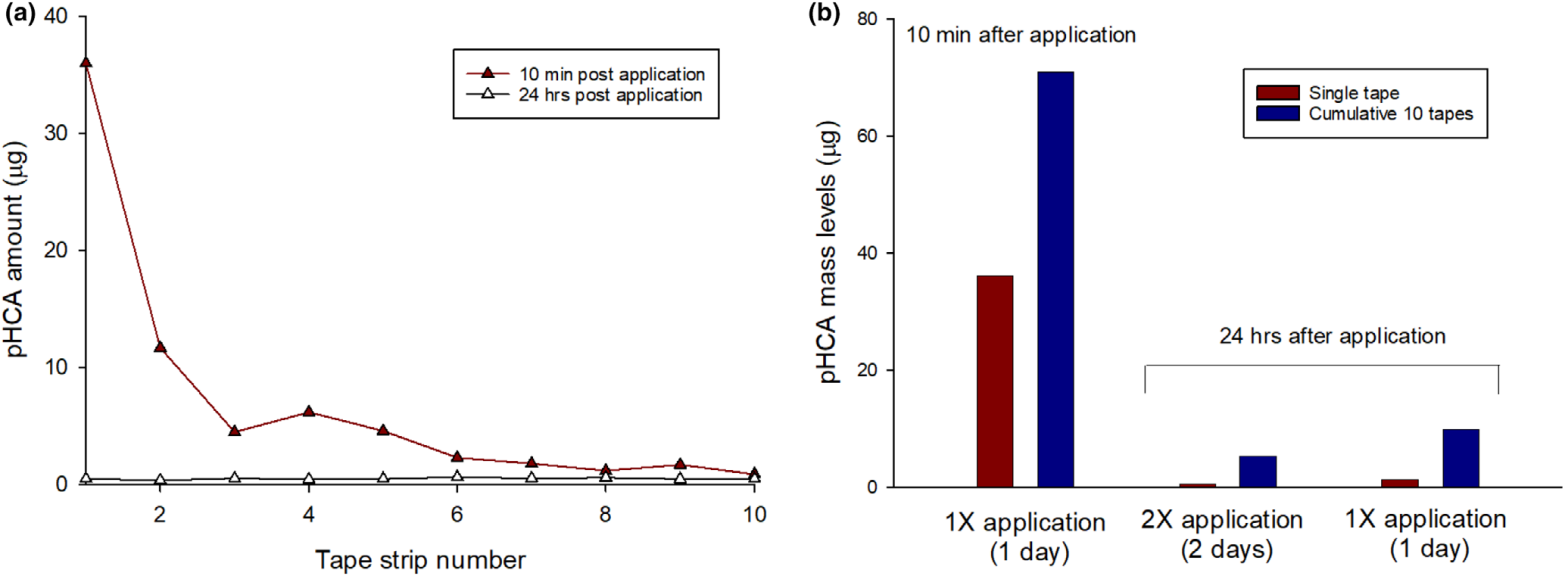
Quantitation of pHCA levels from skin surface after application. (a) pHCA mass levels detected after extraction from each of tapes 1 through 10 sequentially collected from forearm sites 10 min or 24 h after treatment application. (b) The cumulative pHCA mass amounts from 10 tapes collected sequentially 10 min after treatment application shows a 50% increase in levels compared with amount collected from a single tape. Quantitation of pHCA mass levels 24 h after treatment application shows no significant differences in levels between a 1 or 2 day application from a single tape nor cumulative levels from 10 consecutive tapes.

To address the question of whether pretreatment with pHCA led to an accumulation of surface levels that may provide a sunscreen type protection, we performed a separate analytical study that used D‐squame tape strips to sample from skin that have been treated with pHCA after 1 or 2‐day treatment. Analysis of pHCA levels from tapes showed that there is detection of relatively high levels of pHCA 10 min after application (Figure 4a). Further sampling over 10 tapes showed a decrease in detected levels to trace levels. Additionally, sampling 24 and 48 h after application measured 88%–97% lower levels of pHCA levels compared to the levels measured from 1 or 10 cumulative tapes collected 10 min after application (Figure 4b). These results support that there is not a meaningful surface level of pHCA after 2 weeks of pretreatment and that the mitigation of UV‐induced erythema is more biological in nature and not a surface sunscreen‐like artefact.

## DISCUSSION

Cumulative exposure of human skin to environmental stressors is one of the biggest factors that leads to molecular reprogramming and structural changes associated with photoaging. In order to block the negative impact and impact photoaging it is of importance to identify technologies which can provide protection to the skin from acute exposure that accumulates over time. Cinnamate‐based chemistries have been known to absorb UV and have been commercialized to provide a sunscreen level of protection with Octinoxate being a prime example. However, phenolics such as cinnamates from plants have also been known to have biological activity separate from UV absorption properties. pHCA represents one such phenolic cinnamate which is known to have antioxidant and anti‐inflammatory properties.

Previously it has been reported that 1.5% pHCA can mitigate in part UV‐induced damage to skin after pretreatment [11]. Our work provides additional insights to this effect by pHCA in which we evaluated it at two lower concentrations and compared it directly against Octinoxate. Additionally, pHCA was tested in combination with Nam since we had previously shown that Nam can mitigate UV‐induced damage to human skin [14]. It was suggested that additional strategies of combinations with Nam will be needed to enhance these beneficial effects [14]. Finally, we show that pHCA with Nam can partially mitigate induction of inflammation via quantitation of the skin surface biomarker ratio of IL‐1RA/IL‐1α. This mechanistically correlates mitigation of erythema with a lower inflammatory response. The ratio of these cytokines has been previously reported to provide a measure of the underlying inflammatory state of skin in such conditions as acute UV exposure [16, 17] and have also been reported to remain elevated from chronic exposure across [18]. Additionally, the IL‐1α signalling pathway is known to be a significant component of the senescence associated secretory phenotype (SASP) [19]. While an inflammatory response can resolve post‐stress, it has been suggested that this resolution becomes less efficient with aging and leads to a persistent subchronic inflammatory state. This has been proposed as the basis for ‘inflammaging’ that leads to premature aging of tissue and disease [20, 21]. A persistent inflammatory state can also lead to senescence that is hallmarked by the presence of components that make up the senescence associated secretory phenotype SASP [22]. The mitigation of acute UV‐induced IL‐1RA/IL‐α ratio levels by pHCA in combination with Nam supports that pHCA may also play a role in reducing subchronic levels associated with inflammaging as previously reported [14]. This measure of reduced inflammation by pHCA was supported by the correlating mitigation of erythema induced by UV.

Ester derivatives of cinnamic acid such as Octinoxate (octyl methoxycinnamate) have been historically used in sunscreen products as UV filters [23]. Since it has been reported that topical Octinoxate has a relatively low skin penetration profile and found to have low steady state plasma concentrations from topical delivery [24, 25, 26], we used this ester cinnamic acid to assess whether there is any impact on mitigating UV‐induced erythema from 2 weeks of pretreatment in direct comparison to pHCA at equivalent percentage levels based on molecular weight differences (Figure 2d). Interestingly, treatment with Octinoxate did not show any preventative effects after 2 weeks of pretreatment. Overall, we do not believe that the mitigating effect by pHCA against UV‐induced damage was due to physical or chemical absorption of UV by residual surface levels of pHCA because (1) there was no product application on the day of UV exposure, (2) Octinoxate, a cinnamate‐based sunscreen molecule had no effect after 2 weeks of pretreatment, and (3) surface quantitation of pHCA after 1 or 2 days application detected levels that would be below guided amounts for proper SPF protection [27].

The findings presented here show that pHCA in combination with Nam can enhance the effects of mitigating environmental damage from stressors such as UV better than either alone. It should also be noted that pHCA at both 0.3% and 2% appears to have a stronger mitigation effect than 5% Nam across these three studies. These findings support the hypothesis that pHCA alone or in combination with Nam could help mitigate the early signs of premature aging of skin and maintain overall skin homeostasis and health. The work presented here contributes to a previous report of pHCA mitigating UV‐induced damage [11]. The findings from these three studies provides a broader fundamental understanding of pHCA's effects by assessing dose response sensitivity, combinations with Nam, and the reduction of UV‐induced levels of the inflammatory biomarker ratio IL‐1RA/IL‐1α. We also provide evidence that suggests these mitigation effects are not due to residual surface material leading to an artefact of surface absorption. Future work is needed to better understand the mechanistic impact of pHCA on the skin's protective processes at the molecular levels that leads to this mitigation of UV‐induced damage. Overall, the data presented here provides supporting evidence that SASP associated inflammation induced by environmental stressors such as UV can be mitigated by pHCA to help maintain skin's overall homeostasis and delay premature aging.

## CONFLICT OF INTEREST STATEMENT

All authors are employed by The Procter & Gamble Company and report no conflict of interest.
